# A descriptive study of lateral spondylolisthesis in patients with adult scoliosis

**DOI:** 10.1186/1748-7161-7-S1-P13

**Published:** 2012-01-27

**Authors:** P Knott, S Thompson, S Mardjetko

**Affiliations:** 1 Rosalind Franklin University of Medicine and Science, North Chicago, USA; 2 Illinois Bone and Joint Institute, USA

## 

Lateral Spondylolisthesis is seen as a consequence of degenerative disc disease. It occurs primarily in the lumbar spine, and is often associated with adult degenerative scoliosis. When it occurs, it can result in severe back pain from disc instability and radicular leg pain from nerve root compression [1-6].

This is a descriptive study of a series of 32 patients with Lateral Lumbar Spondylolisthesis to evaluate the demographics of the population that this occurs in, the symptoms that it causes, and the association that it has with scoliosis.

All patients seen by the authors in a spinal deformity clinic during the calendar year 2010 had their radiographs screened for evidence of lateral Spondylolisthesis. If the 1319 patients screened, this condition was found in 32 patients. They were included if their Spondylolisthesis was greater than 2mm. The cervical, thoracic and lumbar films were screened, but all 32 patients had their lateral spondylolisthesis in the lumbar spine only.

They were primarily female (84%) and averaged 63 years of age. The youngest patient seen was 35 years old and had a congenital Klippel-Feil Syndrome and congenital scoliosis in the lumbar spine. The others had primarily adult degenerative scoliosis. There was a high prevalence of osteoporosis (41%).

Patients primarily complained of low back pain (94%), but 22% also complained of radicular leg pain. Two patients who did not complain of either leg or back pain both had Down Syndrome. Radiographs showed degenerative scoliosis in all patients, with an average lumbar curve of 50 degrees.
